# Colorectal cancer and gut microbiota studies in China

**DOI:** 10.1080/19490976.2023.2236364

**Published:** 2023-07-23

**Authors:** Zikai Wang, Wanyue Dan, Nana Zhang, Jingyuan Fang, Yunsheng Yang

**Affiliations:** a Microbiota Division, Department of Gastroenterology and Hepatology, The First Medical Center, Chinese PLA General Hospital, Beijing, China; b National Clinical Research Center for Geriatric Diseases, Chinese PLA General Hospital, Beijing, China; c Medical School, Nankai University, Tianjin, China; d Division of Gastroenterology and Hepatology, Renji Hospital, Shanghai Jiao Tong University School of Medicine, Shanghai, China

**Keywords:** Gut microbiota, colorectal cancer, microbial carcinogenesis, microbial biomarkers, anti-tumor therapy, microbiota interventions

## Abstract

Colorectal cancer (CRC) is the third most common malignant tumor worldwide. The incidence and mortality rates of CRC have been increasing in China, possibly due to economic development, lifestyle, and dietary changes. Evidence suggests that gut microbiota plays an essential role in the tumorigenesis of CRC. Gut dysbiosis, specific pathogenic microbes, metabolites, virulence factors, and microbial carcinogenic mechanisms contribute to the initiation and progression of CRC. Gut microbiota biomarkers have potential translational applications in CRC screening and early diagnosis. Gut microbiota-related interventions could improve anti-tumor therapy’s efficacy and severe intestinal toxic effects. Chinese researchers have made many achievements in the relationship between gut microbiota and CRC, although some challenges remain. This review summarizes the current evidence from China on the role of gut microbiota in CRC, mainly including the gut microbiota characteristics, especially *Fusobacterium nucleatum* and *Parvimonas micra*, which have been identified to be enriched in CRC patients; microbial pathogens such as *F. nucleatum* and enterotoxigenic *Bacteroides fragilis*, and *P. micra*, which Chinese scientists have extensively studied; diagnostic biomarkers especially *F. nucleatum*; therapeutic effects, including microecological agents represented by certain *Lactobacillus* strains, fecal microbiota transplantation, and traditional Chinese medicines such as Berberine and Curcumin. More efforts should be focused on exploring the underlying mechanisms of microbial pathogenesis of CRC and providing novel gut microbiota-related therapeutic and preventive strategies.

## Introduction

Colorectal cancer (CRC) threatens the health and causes severe social burdens, accounting for about 10% of all new cancer cases.^1^ Data from the National Cancer Center of China in 2022 showed that CRC ranked fourth in incidence, fifth in mortality among male patients, third in incidence, and fourth in mortality among female patients.^2^ Europe, Oceania, and northern America have the highest CRC incidence and mortality rate globally. However, recent studies showed that the incidence and mortality of CRC in some regions decreased but increased in China.^3^ This trend might be influenced by adopting Western dietary and lifestyle patterns and the accompanying alterations in the gut microbiota.^4^ Improving early diagnosis and treatment and reducing the disease burden of CRC is a significant public health problem. The gut microbiota plays an essential role in CRC occurrence and progression. This review focally addresses Chinese studies on CRC and gut microbiota to describe the characteristics of the intestinal microbiota associated with CRC, the possible microbial mechanisms, the gut microbiota-related biomarkers for screening, early diagnosis, and risk warnings. It also addresses the role of the intestinal microbiota in chemoradiotherapy and immunotherapy and the application of intestinal microbiota interventions with Chinese characteristics, including microecological agents, fecal microbiota transplantation (FMT), and traditional Chinese medicine (TCM).

## CRC-associated gut microbiota profiles

### Intestinal bacterial microbiota and CRC

There are characteristic changes in gut microbiota in various CRC stages, including colorectal adenoma (CRA) and early and advanced CRC.^5–8^ The CRC cohort-specific noises, like ethnic, geographical, and genetic heterogeneity, might distort the structure of gut microbiota dysbiosis and lead to inconsistent conclusions.^5^ Searching for a potential core co-set of microorganisms from different cohorts that might be carcinogenic is of great interest to CRC studies.^6^ Therefore, current studies focused on the gut bacterial microbiota related to CRC across the discovery and validation cohorts based on different ethnic and countries. Unlike CRC patients in Austria, Germany, and France, the gut bacterial composition of CRC patients in China has a unique characteristic with a lower proportion of *Firmicutes* and a higher proportion of *Verrucomicrobia*.^9^ Generally, the characteristics of the intestinal bacterial microbiota of CRC in recent Chinese studies are detailed in Table 1. These findings suggest significant differences in the intestinal bacterial microbiota of Chinese CRC patients with respect to different populations,^10–14^ disease progression^15–19^ and prognosis.^20,21^ In the southern Chinese population, the proportion of intestinal *Fusobacteria* is higher than in Western populations, and CRC in this population may be linked to *F. varium* and other *Fusobacteria* species in addition to *Fusobacterium nucleatum*.^22^ Several studies from China have integrated the gut metagenomic data of CRC patients among Chinese and other ethnic cohorts and found many bacterial genera, including *Fusobacterium*, *Parvimonas*, *Peptostreptococcus*, *Porphyromonas*, *Gemella*, *Prevotella, Solobacterium*, are associated with CRC. CRC-related bacterial species include *F. nucleatum*, *Escherichia coli*, *Bacteroides fragilis*, *P*. *micra*, *P*. *stomatis*, *P*. *ascharolytica*, *P*. *somerae*, *P. intermedia*, *P. anaerobius*, *Clostridium symbiosum*, *S. moorei*, and others.^5, 23–25^ The abundance of *F. nucleatum* changes in the CRA stage and increases in intramucosal carcinoma and advanced CRC; the relative abundance of *Atopobium parvulum* and *Actinomyces odontolyticus* increases significantly multiple CRA and intramucosal cancer stages.^26^ The incidence of early-onset CRC has been increasing globally, which could display adverse clinical and histopathological features. The characteristic changes of gut microbiota associated with early-onset CRC are unclear. A few studies on the Chinese population showed that *Flavonifractor plautii*, *Actinomyces*, and *Schaalia cardiffensis* were the critical microbes in early-onset CRC patients.^27,28^ Moreover, the diversity of the intestinal bacterial microbiota in left-sided colon cancer is higher than in right-sided colon cancer, and the relative abundance of *B. vulgatus* and *C. perfringens* is higher.^29^

**Table 1.** Characteristics of the intestinal bacterial microbiota of CRC in recent Chinese studies.

Authors	Population	Sample collection	Microbial diversity	Significant enrichment	Significant losses
Dai et al.^5^	CRC vs. controls	Feces	Decrease in the Hong Kong cohort	*Bacteroides fragilis, Fusobacterium nucleatum, Porphyromonas asaccharolytica, Parvimonas micra, Prevotella intermedia, and Alistipes finegoldii*	
Xie et al.^10^	CRC vs. controls	Feces		*Clostridium symbiosum* and *F. nucleatum*	
Zhang et al.^11^	CRC vs. controls	Feces	No statistical differences	*Peptostreptococcus stomatis*, *P. micra*, *Gemella morbillorum*, *P. asaccharolytica*, *S. moorei*, *F. nucleatum*, *Ruminococcus torques*, *Campylobacter rectus*, *Clostridium scindens*, and *Caproicibacterium lactatifermentans*	*Eubacterium eligens*, *Eubacterium hadrum*, *Eubacterium hallII*, *Eubacterium desmolans*, *Blautia faecis*, *Roseburia faecis*, *Ruminococcus lactaris*, and *Streptococcus salivarius*
Yu et al.^12^	CRC vs. controls	Feces	Decrease	*P. micra*, *Solobacterium moorei*, and *F. nucleatum*	
Li et al.^13^	CRC vs. HC	Feces		*B. fragilis*	*Faecalibacterium prausnitzii*, *Bifidobacterium adolescent*, *Clostridium clostridioforme*, *Blautia producta* and *R. faecis*
Chen et al.^14^	CRC vs. HC	Feces		The number of viable colonies of *Escherichia coli* and *Enterococcus faecalis*	The number of living colonies of *Campylobacter*, *Bifidobacterium*, and *Lactobacillus*
Yuan et al.^15^	CRC vs. HC	Feces	Decrease	*Bifidobacterium*, *Bacteroides*, and *Megasphaera*	*Collinsella* and *Faecalibacterium*
Chang et al.^16^	CRC vs. HC	Feces	No statistical differences	*G. morbillorum* and *Shigella sonnei*	*Streptococcus gallolyticus*
Du et al.^17^	CRC vs. HC	Feces	Decrease	*Bacteroidota*, *Fusobacteriota*, *and Campylobacter*	*Firmicutes*
Coker et al.^18^	CRC vs. HC	Feces		*P. stomatis*, *F. nucleatum*, *P. micra*, *Peptostreptococcus anaerobius*, and *B. fragilis*	*Eubacterium ventriosum*, *Roseburia interinalis* and *Roseburia inulivorans*
Yeoh et al.^19^	CRC vs. non-CRC	Feces		*Fusobacterium gonidiaformans* and *F. nucleatum*	
Yang et al.^20^	CRC vs. healthy family members	Feces		*Roseburia inulinivorans*, *Clostridium ramosum*, *Porphyromonas gingivalis*, *F. nucleatum*, and *G. morbillorum*	
Li et al.^21^	CRC	Tumor mucosa vs normal mucosa	No statistical differences	*Fusobacterium*, *Gemella*, *Campylobacter*, and *Streptococcus*	*Rhodococcus*, *Blautia*, and *Dorea*
Liu et al.^7^	CRC	Tumor tissues vs. adjacent normal tissues	No statistical differences	*Peptostreptococcus* and *Fusobacterium*	
	CRC vs. CRA	Tumor tissues	Decrease	*Fusobacterium*, *Bacteroides*, *Parvimonas*, and *Prevotella*	
Feng et al.^8^	CRC vs. advanced CRA or HC	Feces	No statistical differences	*A. putredinis*, *Lachnospiraceae bacterium* and *E. coli*	*Streptococcus thermophilus*
Hua et al.^22^	CRC vs. CRA vs. HC	Feces	No statistical differences		*Acidaminococcus* exhibited a decreased relative abundance from HC to CRA and CRC
Gao et al.^23^	CRC vs. CRA vs. HC	Feces			*Firmicutes* exhibited a decreased relative abundance from HC to CRA and CRC
Wu et al.^24^	CRC vs. CRA	Feces	No statistical differences	*F. nucleatum*, *P. asaccharolytica*, *P. stomatis*, *P. micra*, and *Escherichia-Shigella* sp.	*Blautia obeum*, *Butyricicoccus faecihominis*, and *Dorea longicatena*
Jin et al.^25^	CRC vs. CRC postoperative	Feces	No statistical differences	*Gemella, Howardella*, and *Parascardovia*	*Tyzzerella 3* and *Lawsonella*
Huang et al.^26^	CRC vs. HC	Feces	Increase	*Negativicutes* and *Clostridia*	*Clostridia* and *Negativicutes*
	CRC cases after chemotherapy vs. CRC		No statistical differences	*Verrucomicrobia* and *Akkermansiaceae*	
Huo et al.^27^	CRC recurrence vs. non-CRC recurrence	Tumor tissues	No statistical differences	*Firmicutes*, *Bacteroidetes*, and *Fusobacteria*	
		Off-tumor tissues			*Firmicutes* and *Bacteroidetes*
Nakatsu et al.^28^	early-stage CRC vs. late-stage CRC	Lesion-adjacent mucosae	No statistical differences	*Fusobacterium*, *Parvimonas*, *Gemella*, and *Leptotrichia*	*Bacteroides*, *Blautia*, *Faecalibacterium prausnitzii*, *Collinsella aerofaciens* and *Alistipes putredinis*
Xu et al.^29^	young-onset CRC vs. old-onset CRC	Tumor tissues	Decrease	*Schaalia cardiffensis*, *Diaphorobacter*, *Actinomyces*, *Actinomycetaceae*, and *Actinomycetales*	*Acidobacteriota* and *Pyramidobacter piscolens*
Yang et al.^30^	young-onset CRC vs. old-onset CRC	Feces	Increase	*Fusobacterium*, *Flavonifractor*, and *Odoribacter*	*Streptococcus*, *Fusobacterium*, and *Gemella*
Zhong et al.^31^	Left-sided colon cancer vs. right-sided colon cancer	Tumor tissue and feces	Increase	*Clostridium perfringens*, *Bacteroides coprocola* DSM 17,136, *Collinsella aerofaciens*, and *S. gallolyticus* subsp. *Macedonicus* in patients from Harbin	*Bifidobacterium dentium* and *Ruminococcus* sp. 15975 in patients from Harbin
				*Bacteroides vulgatus* in patients from Xiamen	*B. fragilis* and *S. gallolyticus* subsp. *Macedonicus* in patients from Xiamen

CRC: colorectal cancer; CRA: colorectal adenoma; HC: healthy controls.

### Gut mycobiota and viral microbiota in CRC

The enteric mycobiota is an essential component of the intestinal microbiota that is relatively unexplored.^30^ High-throughput sequencing and bioinformatics techniques demonstrated the ecologic association of gut mycobiota with the pathogenesis of human diseases.^31^ Recent studies identified dysbiosis of intestinal fungal microbiota in CRC.^32–34^ The ratio of *Basidiomycota*/*Ascomycota* and the abundance of *Malassezia* are increased, *Saccharomyces* and *Pneumocystis* are decreased, six genera are enriched, and the abundances of 38 species are changed.^33^ *S. cerevisiae* is a probiotic absent in the intestine of CRC patients; previous studies showed that *S. cerevisiae* delays CRC in APC^min/+^ mice.^33,35^ A meta-analysis of seven datasets found that the abundance of six fungi, including *Aspergillus rambelli*, was increased in CRC patients, and subsequent in vitro and in vivo experiments showed that *A. rambelli* promotes the growth of CRC cells in mice.^32^ That study found that intestinal fungal-fungal and fungal-bacterial interactions are significantly enhanced in CRC and positively correlated with the course of CRC.^32^ The SYK/CARD9 signaling pathway protects against CRC by restricting the gut mycobiota-mediated expansion of myeloid-derived suppressor cells.^36^ Studies are needed to clarify the mechanism of intestinal mycobiome participating in the carcinogenesis of CRC.

The enteric virome, an essential component and regulator of gut microflora, affects the intestinal microbiota’s structure and abundance, potentially impacting CRC occurrence, progression, and outcomes by altering bacterial-host communities.^37–39^ The abundance and diversity of gut viral microbiota such as *Siphoviridae*, *Myoviridae*, and *Podoviridae* increase significantly, and *Herelleviridae* is significantly depleted in CRC patients.^37^ Intestinal viral dysregulation is associated with early and late stages of CRC, and viruses such as *Betabaculovirus*, *Punalikevirus*, and *Mulikevirus* were associated with clinical outcomes.^38^ A meta-analysis of metagenomic data showed that bacteriophages of *Porphyromonas*, *Fusobacterium*, and *Hungatella* were enriched in CRC patients.^40^ Another study found that *F. nucleatum*, *Peptacetobacter hiranonis*, and *P. micra* bacteriophages are abundant in CRC patients.^41^ Remodeling the bacteriophage structure might have potential therapeutic value in CRC prevention and treatment. M13 bacteriophage binds to *F. nucleatum* and transforms into M13@Ag, improving the tumor immune microenvironment and inhibiting the proliferation of immunosuppressive myeloid-derived suppressor cells; it acts with the immunosuppressive agent PD-L1 or chemotherapeutic agents to prolong the survival of tumor-bearing mice significantly.^42^ Intestinal bacteriophages have potential applications in screening CRC-related biomarkers and treating drug resistance associated with anti-tumor therapy. The interactions of gut viral microbiota with bacterial microflora and human host and their carcinogenic and anti-tumor effects in CRC need further investigation.

## Intestinal microbial metabolites associated with CRC

The dietary pattern of the Chinese has been plant-based (high in cereal and fiber) in the past few decades. However, the consumption of sugar, fat, and animal-source foods has been increasing among the Chinese population in recent years, which may be associated with an increased incidence of CRC.^43^ Gut microbiota could directly interact with dietary compounds like dietary fiber and produce some metabolites such as short-chain fatty acids (SCFAs) and bile acids (BAs). The change in dietary patterns might alter the gut microbiota and its metabolites. Then, the disrupted gut microbiota-related metabolites contribute to CRC carcinogenesis.^44^

### SCFAs

SCFAs, including butyrate, propionate, and acetate, are metabolites generated from the gut microbial fermentation of insoluble dietary fiber and build connections among dietary patterns, gut microbiota, and intestinal function. Many bacteria, such as *Clostridium* and *Bifidobacterium*, could transform dietary fiber into SCFAs.^45^ SCFAs serve as energy substrates of colonic epithelial cells and maintain intestinal homeostasis through several vital biological processes. For example, SCFAs could regulate the metabolism of colonic epithelial cells, enhance gut barrier function, and modulate the immune response of the intestine, and the activation of G protein-coupled receptors and the inhibition of histone deacetylase activity may be the potential mechanisms.^46^

A high-fiber diet could prevent and reduce the occurrence of CRC, which was associated with the increased abundance of SCFAs-producing bacteria and the raised level of SCFAs in the intestine.^46^ Moreover, a low-fiber diet results in a lack of butyrate-producing bacteria and a lower level of butyrate in the gut observed in CRA and CRC patients. However, the interaction among SCFAs, the gut microbiota, and CRC is complex. SCFAs could antagonize the proliferation, accelerate the apoptosis of CRC cells, and suppress the inflammation-related CRC carcinogenesis pathways.^46^ SCFAs reduce the burden of carcinogens such as BAs by activating drug-metabolizing enzymes and inhibiting the degradation of primary BAs to secondary BAs.^47^ SCFAs also serve as a tumor suppressor to modulate gene expression through epigenetic effects.^48^ In addition, the dysregulation of SCFAs glucose metabolism could be initiated through the intestinal dysbiosis of CRC, and the interaction between SCFAs transporters and glycolysis might correlate with the initiation and progression of CRC.^49^

Gut microbiota-derived SCFAs can be potentially applied in CRC prevention and treatment. Supplementation of dietary fiber or postbiotics containing SCFAs might play an essential role in CRC prevention;^50^ the appropriate formulation and amounts and the efficacy evaluation should be further investigated. Furthermore, several preclinical studies showed that SCFAs-producing bacteria and butyrate could enhance the efficacy of chemotherapy drugs such as oxaliplatin and irinotecan and improve the response to immunotherapy;^51^ and then SCFAs could alleviate the adverse effect caused by chemotherapeutic adverse effects such as colitis. Studies based on animal models showed the potential value of intestinal microbiota-derived SCFAs in CRC.^52^ In-depth investigations are needed before the clinical transformation and application of SCFAs in CRC prevention and treatment.

### BAs

Individuals who consume high-fat diets have a higher incidence of CRC. Western diets, including high-fat, red, and processed meat, increase the level of fecal secondary BAs, mostly deoxycholic acid and lithocholic acid. The secondary BAs metabolized by gut microbiota are carcinogenetic factors in CRC development.^53^ *Clostridium* and *Eubacterium* mediate BA-related 7α-dehydroxylation and sulfidation. Elevated levels of secondary BAs exert detrimental tumor-promoting effects via mechanisms such as colonic epithelial barrier function injury, oxidative damage to DNA, inflammation, activation of NF­κB signaling pathway, and increased cell proliferation.^54^ Studies also showed that cholecystectomy might be associated with CRC, which may be due to the disruption of BAs secretion and intestinal microbiota imbalance.^55^ BAs modulate the intestinal microbiota, which regulates the BA pool; the disruption of BA-gut microbiota crosstalk contributes to CRC development. However, the synthesis, transport, and metabolism of BAs and the mechanisms of BA-gut microbiota interactions involved in carcinogenesis remain unclear and should be further investigated. Moreover, the modulation of gut microbiota, BAs, and BA-related receptor signaling pathways (such as farnesoid X receptor and G protein-coupled bile acid receptor 1) might be novel therapeutic targets and research frontiers for CRC.^56^

### Hydrogen sulfide (H_2_S)

H_2_S is generated from the degradation of food-derived nutrients in the intestine by sulfate-reducing bacteria (SRB) such as *Desulfovibrio* spp. H_2_S promotes CRC occurrence and development by damaging intestinal epithelial cells, inducing free radical release, DNA damage, and colonic mucosal inflammation, inhibiting butyrate oxidation, cytochrome oxidase, and DNA methylation^57^. SRB-related intestinal microbial sulfur metabolism is a potential trigger of CRC. A high-fat and high-protein diet can promote the growth of SRB, metabolizing a large amount of genotoxic H_2_S related to CRC progression.^58^ There are elevated intestinal H_2_S levels in CRC patients, and endogenous and microbial H_2_S produced by cysteine at CRC sites show an upward trend.^59^

## The microbial pathogenesis of CRC

**Figure 1.**
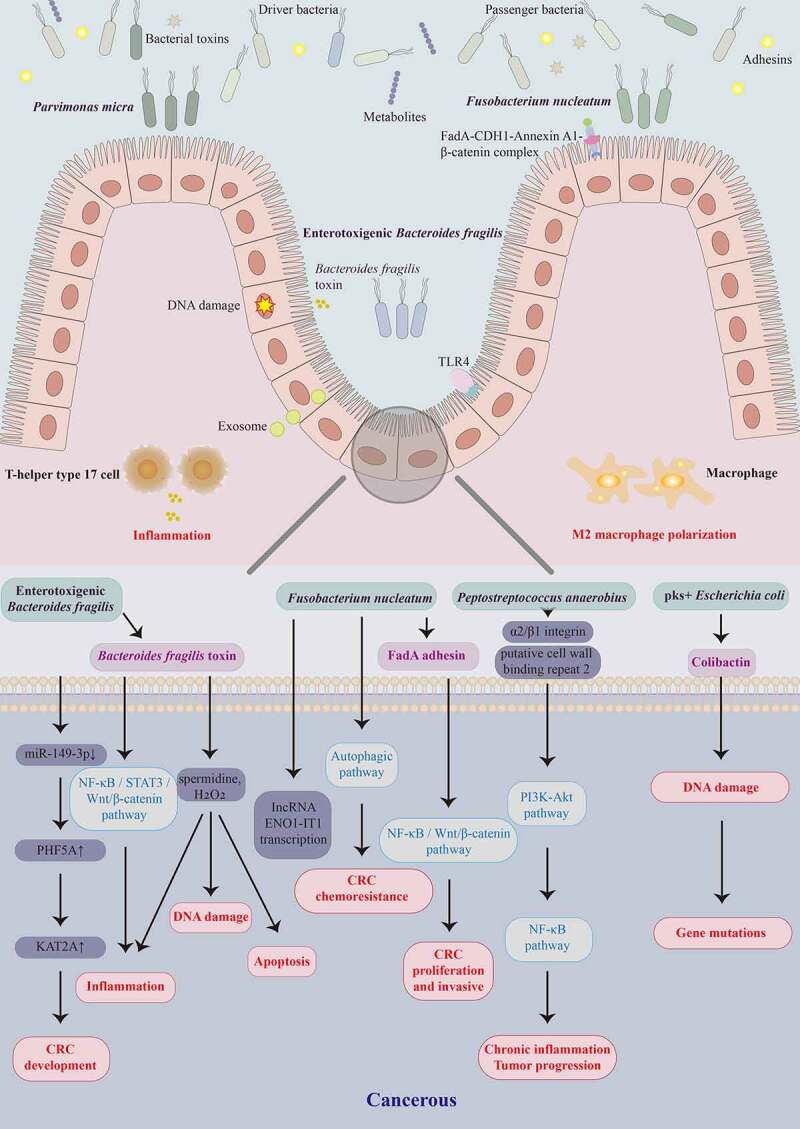
Schematic representation of gut microbiota and their metabolites involved in colorectal carcinogenesis at the cellular and molecular levels. Gut bacteria such as *Fusobacterium nucleatum*, enterotoxigenic *Bacteroides fragilis*, *Peptostreptococcus anaerobius*, pks^+^ *Escherichia coli*, and *Parvimonas micra*, and their virulence factors FadA, *B. fragilis* toxin, and colibactin contribute to the colorectal cancer (CRC) development by activating different pathways that trigger DNA damage, intestinal inflammation, macrophage polarization, and apoptosis.

CRC carcinogenesis involves genetic and environmental factors. Genetic mutations, epigenetic changes, inflammation, immune regulation, and metabolic and hormonal disorders contribute to CRC. The main pathway in colorectal carcinogenesis is the “adenoma-carcinoma sequence,” which describes that most CRC cases are sporadic and progress slowly from normal to dysplastic epithelium to carcinoma throughout years.^60^ Besides, the gut microbiota is the essential environmental factor contributing to the initiation, progression, metastasis, and CRC outcomes. The intestinal microbiota of CRC patients promotes colorectal dysplasia and tumor formation in a germ-free mouse model, providing evidence for the gut microbiota’s involvement in CRC progression.^61^ According to available studies, the gut microbiota-related carcinogenic mechanisms of CRC involve an imbalance of intestinal microflora, invasion and colonization of pathogenic microbes, and impaired intestinal barrier function induced by microbial metabolites and virulence factors. The tumor microenvironment is altered by inducing the secretion of pro-inflammatory cytokines and chronic inflammation surrounding intestinal epithelial cells, activating the Wnt/β-catenin and NF-κB pro-inflammatory signaling pathways and exacerbating intestinal epithelial cell DNA damage, ultimately initiating CRC progression (summarized in Figure 1).

There are two models (“alpha-bug” and “driver-passenger”) of intestinal microbial carcinogenesis related to CRC.^62^ The alpha-bug model holds *Helicobacter pylori* in gastric cancer and hepatitis B virus in primary liver cancer stimulates the host to produce inflammatory and immune responses, proto-oncogene activation, and tumor suppressor gene inhibition.^62^ Enterotoxigenic *B. fragilis* (ETBF) -related enterotoxin compromises intestinal epithelial permeability, activation of several tumor-initiating relevant Wnt/β-catenin and STAT3 pathways, and immune responses from persistent Th17 activation, leading to CRC progression.^6^ The abundance of these so-called promoter bacterial microbes significantly increases in cancer or para-cancerous tissues; however, they appear to decrease during CRC progression. The alpha-bug model does not explain the increased abundance of undifferentiated gut microflora in the early stage of CRC progression. Therefore, a driver-passenger model was proposed to explain the complex changes in the intestinal microbiota associated with CRC.^63^ For example, *E. coli* and ETBF would be driver bacteria that induce DNA damage in intestinal epithelial cells and trigger inflammation to initiate carcinogenesis. These organisms form a unique tumor microenvironment conducive to increasing opportunistic pathogens called passenger bacteria. These passenger bacteria, such as *Streptococcus bovis*, *S. gallolyticus*, *Roseburia*, and *Fusobacterium*, might participate in cancer promotion and even competitively inhibit driver bacteria growth. On the other hand, the traditional “adenoma-carcinoma sequence” is the primary pathway for CRC occurrence and development, and the other pathway of colorectal serrated lesions;^60^ the former involves APC, KRAS, BRAF, TP53, SMAD4, and other gene mutations, while the latter involves CpG island methylation phenotype, MLH1, MGMT, and MSI.^64^

Research has demonstrated that specific “oncomicrobes” promote CRC initiation and progression, with *F. nucleatum*, ETBF, pks^+^ *E. coli*, and *P. micra* being extensively studied.^6^ *F. nucleatum* activates the TLR4/KEAP1/NRF2 axis and the NF-κB signaling pathway, accompanied by inducing M2 macrophage polarization and macrophage infiltration in the tumor microenvironment to promote tumor growth.^65^ In light of this evidence, a “two-hit” model has been proposed with pro-carcinogenic features that may initiate CRC development, in which somatic mutations are the first hit and *F. nucleatum* is the second, exacerbating CRC development after benign cells become cancerous.^66^ Several Chinese research teams contribute to elucidating the gut microbiota-mediated pathogenesis of CRC. Fang et al. from Shanghai found that *F. nucleatum* could activate IncRNA ENO1-IT1 transcription to promote CRC initiation and development and activates the autophagic pathway to promote CRC chemoresistance,^67,68^ while enterotoxigenic *B. fragilis* promotes Th17 cell differentiation through downregulation of miR-149-3p. The team proposed for the first time that gut microbiota and host co-metabolism regulate the gut immune microenvironment. The gut microbiota promotes colorectal carcinogenesis by mediating the host urea cycle and disrupting intestinal immune homeostasis.^69^ Furthermore, Yu et al. from Hong Kong focused on the microbial-mediated pathways in CRC. *P. micra* induces tumors in mice by inducing colorectal cell proliferation and modulating the Th17 immune response,^70^ while *P. anaerobius* promotes colorectal tumorigenesis by binding to integrin α_2_/β_1_ and activating NF-κB signaling pathway.^71^

Additionally, many studies focused on the characteristics of the intestinal microbial virulence factors associated with CRC. Gut dysbiosis is carcinogenic through intestinal microbial-associated virulence factors that induce inflammation or immunosuppression. The genetic toxicity of intestinal microbial-associated virulence factors leads to host DNA damage, also involved in CRC initiation and development. The unique FadA adhesin secreted by *F. nucleatum* binds to E-cadherin (CDH1) and forms a FadA-CDH1-Annexin A1-β-catenin complex that activates Wnt/β-catenin signaling to promote initiation and progression of CRC.^72,73^ *F. nucleatum* adheres to E-cadherin on the surface of CRC cells and activates the Wnt/β-catenin and NF-κB inflammatory pathways, increasing the proliferation, and invasive activities of CRC cells, promoting metastasis.^74^ The Fap2 protein of *F. nucleatum* directly interacts with TIGIT, leading to the bacterium-dependent tumor immune evasion mechanism.^75^ ETBF, a subtype of *B. fragilis*, secretes *B. fragilis* toxin (BFT) to activate the NF-κB, STAT3, and Wnt/β-catenin signaling pathways in colonic epithelium cells, triggering inflammation responses and causing excessive cell proliferation; in this way, ETBF promotes carcinogenesis in CRC.^76^ BFT produces spermidine and H_2_O_2_ as by-products of polyamine catabolism, leading to apoptosis, inflammation, and DNA damage.^77^ Colibactin produced by pks^+^ *E. coli* strain induces DNA double-strand breaks in host cells through its deoxyribonuclease activity, activating DNA damage signaling cascades and increasing the frequency of gene mutations associated with CRC development.^78^ Nevertheless, the microbial-associated pathogenesis of CRC and CRA remains to be elucidated. Notably, the research into the pathogenesis of CRC is not limited to several novel pathogenic microbes and their virulence factors to promote CRC initiation and development but continues to advance and deepen.

## Gut microbiota-related biomarkers for the screening and early CRC diagnosis

Although the incidence rate of CRC in China is lower than that in Europe and northern America, the number of CRC patients is the largest globally for the large population.^3^ Most CRC patients are in the middle or late stage of diagnosis and lose the opportunity for surgery, leading to poor outcomes and high mortality rates. The overall burden for CRC diagnosis and treatment remains severe globally. The recent China guideline for the screening and early detection of CRC has recommended that individuals with a high risk of CRC should undergo screening at 40 years of age, and the optimal age for starting CRC screening is 45 years old.^79^ Colonoscopy is regarded as the gold standard for CRC screening, which could reduce the incidence and mortality of CRC.^80^ However, as an invasive procedure, colonoscopy is challenging as a large-scale screening method.^81^ The coverage and participation rate were also lower in China than in America, which limited the effectiveness of nationwide CRC screening.^3^ In addition, fecal occult blood testing, fecal immunochemical test (FIT), and blood DNA methylation biomarkers are the recommended noninvasive screening methods, which are superior to colonoscopy regarding clinical compliance and cost-effectiveness; nevertheless, they still have limited sensitivity for advanced CRA and CRC.^82^ Longitudinal studies involving various stages of CRC and cross-sectional studies of different CRC cohorts suggested that the intestinal microbiota is specific in CRC and its precancerous lesions.^5,24,83^ This finding provides direct evidence for the potential role of microbial biomarkers in CRC screening and early detection. With the development of multi-omics and bioinformatics artificial intelligence algorithms, innovating noninvasive intestinal microbiota-related biomarkers have become an essential research field in CRC screening and early diagnosis. The intestinal microbiota-related biomarkers for the screening and early diagnosis of CRC in recent Chinese studies are presented in Table 2.

**Table 2.** Gut microbiota-related biomarkers for the screening and early diagnosis of CRC in recent Chinese studies.

Authors	Cohort	Gut microbiota-related biomarkers	AUC
**Bacterial-related biomarkers**			
Gao et al.^23^	CRC vs. CRA	the combined panel of 12 species (*Parvimonas* unclassified, *Fusobacterium nucleatum*, *Roseburia intestinalis*, *Gardnerella vaginalis*, *Peptostreptococcus anaerobius*, *Peptostreptococcus stomatis*, *Lactobacillus iners*, *Gemella morbillorum*, *Porphyromonas asaccharolytica*, *Faecalibacterium prausnitzii*, *Atopobium vaginae*, and *Parvimonas micra*) and three metabolites	0.994
Coker et al.^18^	CRC vs. NC	11 metabolite biomarkers and six bacterial species (*F. nucleatum*, *P. anaerobius*, *P. micra*, *Roseburia inulinivorans*, *Eikenella corrodens*, and *Xanthomonas perforans*)	0.9417
Yang et al.^20^	CRC vs. NC	gene 8,122,329 (unknown function from *Coprobacillus*)	0.930
Chen et al.^85^	CRC vs. CRA	Eight gut microbiome-associated serum metabolites	0.92
Wu et al.^24^	CRC vs. CRA	24 differential biomarkers (*Streptococcus thermophilus* TH1435, *P. micra*, *Bacteroides dorei*, *Clostridium scindens*, *Erysipelatoclostridium ramosum*, *Blautia* sp., *Eubacterium coprostanoligenes* group sp., *Lachnospira pectinoschiza*, *Ruminococcaceae* UCG-002 sp., *Ruminococcus bromii*, *Porphyromonas* sp. HMSC077F02, *Porphyromonas* sp. 2007b, *Eubacterium ruminantium*, *Tyzzerella* 3 sp., *Hungatella hathewayi* WAL-18680, *Blautia faecis*, *Bacteroides nordii*, *Lachnospiraceae* UCG-010 sp., *Eubacterium ventriosum* group sp., *Streptococcus infantarius*, *R. intestinalis*, *Merdibacter massiliensis*, *Ruminococcus gnavus* group sp., *Roseburia hominis* A2–183) with age and body mass index	0.89
Xie et al.^10^	CRC vs. NC	Combination of *Clostridium symbiosum* and *F. nucleatum* abundance with FIT and CEA test	0.876
Hua et al.^22^	CRC vs. CRA	Combination of the top ten species (*Butyricimonas synergistica*, *Agrobacterium larrymoorei*, *Bacteroides plebeius*, *Lachnospiraceae bacterium feline* oral taxon 001, *C. scindens*, *Prevotella heparinolytica*, *bacterium* LD2013, *Streptococcus mutan*s, *L. bacterium* 19gly4, and *Eubacterium hallii*)	0.8554
Yu et al.^12^	CRC vs. NC	Two gene biomarkers (from *F. nucleatum* and *P. micra*)	0.84
Dai et al.^5^	CRC vs. NC	7 CRC-enriched bacterial biomarkers (*B. fragilis*, *F. nucleatum*, *P. asaccharolytica*, *P. micra*, *Prevotella intermedia*, *Alistipes finegoldii*, and *Thermanaerovibrio acidaminovorans*)	0.80
Liang et al.^86^	CRC vs. NC	A simple linear combination of four bacteria (*F. nucleatum* + *Clostridium hathewayi* + undefined species (labeled as m7) - *Bacteroides clarus*)	0.886
**Fungal-related biomarkers**			
Coker et al.^36^	CRC vs. NC	Fourteen fungal biomarkers (*Aspergillus flavus*, *Kwoniella mangrovensis*, *Pseudogymnoascus* sp. VKM F-4518, *Debaryomyces fabryi*, *Aspergillus sydowii*, *Moniliophthora perniciosa*, *Kwoniella heavenensis*, *Aspergillus ochraceoroseus*, *Talaromyces islandicus*, *Malassezia globosa*, *Pseudogymnoascus* sp. VKM F-4520, *Aspergillus rambelli*, *Pneumocystis murina*, and *Nosemia apis*)	0.93
Gao et al.^37^	CRC vs. NC	Thirteen fungal biomarkers (*Phanerochaete chrysosporium*, *A. flavus*, *Entomophthora muscae*, *A. rambelli*, *fungal* sp ARF18, *Metschnikowia cubensis*, *Fusarium pininemorale*, *Spraguea lophii*, *Candida versatilis*, *Lachancea waltii*, *Taxomyces andreanae*, *Exophiala mesophila*, and *Brettanomyces anomalus*)	0.757
**Viral-related biomarkers**			
Nakatsu et al.^41^	CRC vs. NC	Twenty-two viral biomarkers (*Orthobunyavirus*, *Inovirus*, *Tunalikevirus*, *L5likevirus*, *Phikzlikevirus*, *Betabaculovirus*, *Sp6likevirus*, *Sfi21dtunalikevirus*, *Punalikevirus*, *Lambdalikevirus*, *C2likevirus*, *Mulikevirus*, *Twortlikevirus*, *γ-Sphaerolipovirus*, *Circovirus*, *Spounalikevirus*, *Cytomegalovirus*, *Epsilon15likevirus*, *N15likevirus*, *Phikmvlikevirus*, *Cyprinivirus*, and *Lymphocryptovirus*)	0.802
**Multi-kingdom biomarkers**			
Lin et al.^35^	CRC vs. NC	Five fungi (*Aspergillus kawachii*, *Rhizophagus clarus*, *Baudoinia panamericana*, *A. rambelli*, *Trichoderma atroviride)* and nine bacteria (*P. micra*, *F. nucfeatum*, *G. morbillorum*, *Porphyromonas asaccharolytica*, *Dialister pneumosintes*, *Anaerostipes hadrus*, *Romboutsia ilealis*, *Brachyspira pilosicoli*, and *Streptococcus safivarius*)	0.9002
Liu et al.^9^	CRC vs. NC	11 bacterial (*F. nucleatum*, *P. micra*, *G. morbillorum*, *Ruminococcus bicirculans*, *R. intestinalis*, *Pseudobutyrivibrio xylanivorans*, *Streptococcus anginosus*, and *Eubacterium eligens*), four fungal (*A. rambelli*, *Sistotremastrum suecicum*, *T. islandicus*, and *Aspergillus niger*) and one archaeal (*Pyrobaculum arsenaticum*) biomarkers	0.83

AUC: area under the receiver-operating characteristics curve; CRC: colorectal cancer; CRA: colorectal adenoma; NC: normal controls; rpoB: RNA polymerase subunit β; FIT: fecal immunochemical test; CEA: carcinoembryonic antigen.

### Bacterial biomarkers

Several recent studies have focused on gut microbial-related biomarkers for noninvasive diagnosis of CRC and its precancerous lesions,^24,83–85,^ with significant studies by Chinese scientists. *F. nucleatum* is the leading candidate bacterial biomarker for early diagnosis, risk, and outcomes prediction.^84,85^ *F. nucleatum* abundance in patients with CRC and its precancerous lesions was significantly higher than in the control group, and the level of *F. nucleatum* was more elevated in cancer and para-cancerous tissues in CRC.^86^ Changes in the ratio of *F. nucleatum* to *Faecalibacterium prausnitzii* and *Bifidobacterium* in stool samples from CRC patients might identify early CRC.^84^ A Chinese study found that detecting *F. nucleatum* DNA in oral saliva was superior to traditional tumor markers such as CEA and CA-199 in diagnosing CRC, and *F. nucleatum* DNA level was related to overall survival and was an independent factor for predicting CRC outcomes.^87^ Subsequently, quantitative PCR analysis in another Chinese cohort identified *F. nucleatum*-related butyryl-CoA dehydrogenase and *P. micra*-related *rpoB* as discriminators of CRC patients with an area under the ROC curve (AUC) of 0.84; these genes were enriched in patients with early CRC, suggesting that bacteria-related gene markers have the potential for early CRC diagnosis.^24^ Compared with the culture and identification of specific microbial strains, the quantitative analysis of CRC-specific bacterial genes might improve the sensitivity and specificity of CRC screening and early diagnosis, reducing the cost and presenting higher clinical operability. For the bacterial gene marker m3 based on *F. nucleatum*, *Lachnochlostridium* sp., and *C. hathewayi*, the sensitivity and specificity for distinguishing CRA were superior to *F. nucleatum* or the FIT method. The m3 gene combined with *F. nucleatum*, *C. hathewayi*, *B. clarus*, and FIT had a higher diagnostic ability for CRC.^88^ Furthermore, Fang et al. found that FIT combined with quantitative PCR detection of fecal *F. nucleatum* DNA improved the sensitivity and specificity in diagnosing and predicting CRC.^89^ *F. nucleatum* IgA and IgG antibody-based assays combined with traditional tumor markers such as CEA and CA-199 are also used for CRC screening.^90^ Combining gut microbiota-related biomarkers with conventional approaches such as FIT to improve the efficiency of screening and early diagnosis for CRC is essential to the clinical translational application of gut microbiota.^88,89^

In addition to *F. nucleatum*, other intestinal bacterial biomarkers are used for screening for CRA and early CRC. Some studies analyzed the intestinal metagenomic characteristics of CRA, early to advanced CRC at different stages, and revealed characteristic changes of the intestinal microbiota at the precancerous stage of CRC.^26^ Fang et al. validated a set of gut microbiota-based diagnostic models developed to diagnose laterally spreading tumors, the primary precursor lesions of CRC, with an AUC of 0.92.^91^ Besides, Yu et al. identified four CRC-enriched bacterial-related genes in Chinese exploratory and Danish validation cohorts and further validated them in French and Australian populations with AUCs of 0.72 and 0.77, respectively.^24^ *A.parvulum* and *A.odontolyticus* were only significantly increased in multiple CRA and intramucosal carcinoma, suggesting that there are specific microbial biomarkers for early screening of CRC precancerous lesions.^26^ A random forest classifier was constructed with 11 bacterial phylotypes to distinguish CRA from the control group. A classifier with 26 bacterial phylotypes was constructed to distinguish CRC from CRA with an AUC of 0.89; this classifier was validated in two independent cohorts with AUCs of 0.78 and 0.84, respectively, and these findings were confirmed in a new cohort using real-time quantitative PCR.^83^ In addition to screening and early diagnosis of CRC, Wei et al. also validated a set of gut microbiota-based diagnostic models to distinguish the newly developed adenomas from the healthy subjects, which may help prevent cancer recurrence in postoperative patients.^92^

In recent years, multi-omics research showed the potential of intestinal microbial metabolites in CRC screening and early diagnosis.^43,93,94^ One study developed an intestinal microbiome-related eight serum metabolites panel to predict CRC and CRA with an AUC of 0.92 in the validation cohort, which was significantly better than tumor markers such as CEA, showing the potential application value of serum metabolites associated with intestinal microbiota in identifying CRC and CRA.^93^ Through fecal metagenomic and metabolomic analysis of CRA and CRC, at different stages from CRA to CRC, Yu et al. found that norvaline and myristic acid showed an increasing trend, and 20 CRC-enriched metabolites distinguished CRC from CRA and healthy control; further integration of microbial metagenomic data showed that the ability to distinguish CRC from CRA and healthy control was improved with AUC of 0.94 and 0.92 respectively.^44^ Moreover, a few studies from China tried to find the gut microbial biomarkers for early-onset CRC.^27,28,94^ One study showed that multi-omics signatures of early-onset CRC might be associated with enriched *F. plauti* and increased tryptophan, BAs, and choline metabolism. They constructed a microbiome-derived predictive model based on metagenomic, metabolomic, and KEGG orthology gene markers to detect early-onset CRC.^94^ Gut microbial-associated biomarkers have shown significant advantages in the noninvasive diagnosis of CRC, although further validation studies are needed before these biomarkers can be used in clinical practice.

### Viral and fungal biomarkers

Several studies focused on the gut virome- and mycobiome-associated biomarkers for CRC.^33,38,39^ The virome biomarker, including 22 viral metagenomic taxa, distinguished CRC patients from control subjects across discovery and validation cohorts (AUC = 0.8).^38^ Moreover, significant changes in intestinal mycobiome were associated with CRC, with an increased abundance of *Malassezia* and decreased *Saccharomycetes* and *pneumocystis*. Fourteen fungal microbes distinguished CRC with an AUC of 0.93.^33^ Combining fungal and bacterial biomarkers might be more potent for diagnosing CRC; combining *A. rambelli* and *F. nucleatum* improved diagnostic accuracy by 1.4%–10.6%.^32^

The specificity and sensitivity of biomarkers identified from the intestinal microbiota are superior to existing clinical methods for CRC screening and early diagnosis. The gut bacterial, fungal, and viral biomarkers for CRC have the potential for clinical translational application; nevertheless, further investigation is needed. Integrating and optimizing intestinal microbiota-related biomarker panels with traditional clinical methods can improve CRC screening and diagnosis levels.

## Gut microbiota and anti-tumor therapy in CRC

Intestinal microbiota dysbiosis and specific pathogenic microbes have carcinogenic and pro-tumorigenic effects. Substantial evidence shows that the gut microbiota is associated with the efficacy or adverse effects of anti-tumor treatments such as chemoradiotherapy and immunotherapy.^95^ The gut microbiota is a potential biomarker to predict anti-tumor responses and adverse reactions. The gut microbiota regulates the efficacy of anti-tumor therapy and clinical CRC outcomes.

### Chemotherapy

The intestinal microbiota influences the efficacy of chemotherapy, drug resistance, and intestinal-associated toxicity. The gut microbiota mediates the anti-tumor effects of chemotherapeutic drugs (e.g., 5-FU, gemcitabine, and platinum) through various mechanisms, including translocation, immunomodulation, metabolism, enzymatic degradation, reduced diversity, and ecological variation.^96^ The gut microbiota is related to the efficacy of chemotherapy for CRC.^97,98^ Patients with CRC who respond to chemotherapy have a relatively high abundance of *Sutterella* and *Roseburia*; a high abundance of *Fusobacterium* indicates non-response and poor clinical outcomes.^99,100^ Moreover, the gut microbiota might be involved in chemotherapy resistance. *F. nucleatum* is enriched in tumor tissues of recurrent CRC patients after chemotherapy, and the abundance of *F. nucleatum* negatively correlates with the CRC outcomes. *F. nucleatum* induces drug resistance to platinum and 5-FU in CRC by affecting tumor cell autophagy.^68^ A study showed that metronidazole reduced the amount of *F. nucleatum* and shrank the tumor body, suggesting the application of anti-*F. nucleatum* before chemotherapy in *F. nucleatum*-positive CRC patients might enhance chemotherapeutic efficacy.^101^ *F. nucleatum* is emerging as a microbial biomarker for predicting chemotherapeutic efficacy, drug resistance, and outcomes and is becoming a therapeutic target for CRC. The composition and function of the gut microbiota are altered by cytotoxic chemotherapeutic agents, further affecting microbial-associated drug metabolism.^102^ The extent of tumor regression in tumor-bearing mice treated with platinum-based chemotherapy is significantly lower after antibiotic treatment, and germ-free mice do not respond to platinum treatment.^103^ Platinum induces reactive oxygen species (ROS) aggregation, the primary anti-tumor mechanism of platinum. However, no apparent ROS aggregation was observed in germ-free mice or tumor-bearing mice models treated with antibiotics, suggesting that the intestinal microbiota is involved in platinum-induced DNA damage and tumor cell apoptosis.^104^ The intestinal microbiota assists platinum in activating tumor-related inflammatory cells to generate ROS, enhancing the role of platinum in killing tumor cells.^103^ The influence of intestinal microbial-related metabolites on chemotherapy should not be ignored. For example, butyric acid enhances the anti-tumor cytotoxic effect of CD8^+^T cells in vitro and promotes the anti-tumor effect of platinum-based chemotherapeutic drugs.^51^ Supplementation of probiotics alleviates 5-FU and platinum-related intestinal injuries and adverse reactions.^105^ *Lactobacillus* supplementation during chemotherapy in CRC patients treated with 5-FU reduces mortality and improves gastrointestinal symptoms such as diarrhea.^106^ Furthermore, the gut microbiota modulates irinotecan’s metabolism and adverse effects. Irinotecan is a prodrug of SN-38 used to treat CRC. The incidence of irinotecan-related grade 3–4 diarrhea is 20%-40%, which affects its anti-tumor efficacy. SN-38 is glucuronidated to SN-38 G by liver enzymes and excreted to the intestine, where intestinal bacterial β-glucuronidase hydrolyzes SN-38 G to SN-38, resulting in intestinal mucosal injury and diarrhea.^96,107^ Probiotic supplementation improves diarrhea and intestinal mucosal damage associated with irinotecan treatment.^108^ The development of intestinal microbiota biomarkers for predicting the efficacy of CRC chemotherapy and microbial interventions for improving the chemotherapeutic efficacy and adverse reactions need to be further studied.

### Radiotherapy

The intestinal microbiota might play a specific role in CRC radiotherapy, influencing radiotherapy’s efficacy and participating in adverse intestinal events such as mucositis and radiation enteritis. Radiotherapy causes significant variation in the structure, diversity, and abundance of the intestinal microbiota, characterized by decreased abundance of *Firmicutes* and an increased abundance of *Proteus*, *Ackermann*, *Fusobacteria*, and *Bacteroides*.^102^ The abundance of pathogenic microbes in advanced CRC patients after radiotherapy is significantly reduced, while the abundance of beneficial microbes such as *Lactobacillus* and *Streptococcus* is increased. The increase of beneficial bacteria may be related to the efficacy of radiotherapy. Bacterial taxa such as *Thermi* and *Sphingomonadaceae* identify patients with advanced rectal cancer who have achieved complete pathological response and non-pathological complete response after neoadjuvant chemoradiotherapy.^109^ Oral microbiota alterations influence the gut bacterial composition within CRC tumors, and oral bacteria such as *F. nucleatum* migrate to the CRC locus and impair radiotherapy’s therapeutic efficacy and outcomes.^110^

On the other hand, exposure to high-energy radiation causes intestinal mucosal barrier damage and intestinal crypt cell apoptosis, leading to intestinal dysbiosis and increasing the risk of radiation enteritis.^102^ After radiotherapy, the high abundance of *Clostridia*, *Rothia*, and *Koalas* might be related to intestinal epithelium inflammatory reactions and barrier dysfunction, which lead to radiation enteritis.^111^ Regulating intestinal microbiota by supplementing probiotics and FMT might effectively prevent and treat radiation enteritis.^112^ *Bifidobacterium*, *Acidophilus*, *Streptococcus*, and *L. casei* alleviate the severity of radiation enteritis.^113^ The intestinal microbiota is expected to be an interventional target for radiation enteritis.

### Immunotherapy

Immunotherapy has been used in hematologic malignancies and immune-sensitive tumors. Immune checkpoint inhibitors (ICIs) have been explored in treating CRC in a subset of CRC patients with MSI-H phenotype.^114^ For metastatic patients with specific subtypes such as MSI-H or DNA mismatch repair deficiency, immunotherapy can achieve a more sustained therapeutic response,^115^ and combined use of ICIs prolongs survival in patients with advanced refractory CRC.^116^ Improving the curative effect and reducing intestinal toxicity is a critical problem in CRC immunotherapy.

Cancer immunotherapy involves the gut microbiota, which plays an essential role in the complex interactions among tumors, the immune system, and ICIs.^117^ The intestinal microbiota is associated with the efficacy of CRC immunotherapy. ICIs did not inhibit CRC progression in germ-free mice, and the antibiotic treatment weakened the anti-tumor effect of ICIs in mice with normal intestinal microflora.^117^ The effectiveness of ICIs depends on intestinal microbes such as *A. muciniphila*, *B. fragilis*, *Bifidobacterium* sp., *Eubacterium limosum*, *Faecalibacterium* sp., *Prevotella* sp., and *Alistipes shahii*. A preclinical study showed that *A. muciniphila* and *Prevotella* sp. might play an essential role in maintaining the efficacy of ICIs.^117^ The abundance and positive detection of the *Fusobacterium* in non-responders were significantly higher than in responders when combining regofinil with treprizumab to treat refractory and advanced CRC.^100^ *B. fragilis* induces dendritic cells to mature and helper T lymphocytes to produce an immune response, then enhance the efficacy of ICIs. *Bifidobacterium* enhances the killing effect of CD8^+^T cells on tumors by changing the activity of dendritic cells. The gut microbiota is indispensable for the immune response of immunotherapy, which promotes host immunity, improves the host immune microenvironment, and affects the antigen presentation process, thereby changing the outcome of immunotherapy.^118^

Intestinal microbiota dysbiosis is involved in ICIs-associated colitis.^119^ Increases in *Bacteroidetes* are associated with resistance to ICIs-associated colitis; polyamine transport and vitamin B synthesis pathways are associated with colitis predisposition.^120^ Intestinal microbiota interventions improve metabolic pathways and immunotherapy’s efficacy.^121^ A set of 11 commensal bacterial strains enhance the therapeutic efficacy of ICIs in syngeneic tumor models.^122^ Understanding the mechanisms of the intestinal microbiota in promoting ICIs efficacy or drug resistance, exploring microbiota-related biomarkers, enhancing ICIs efficacy, and treating ICIs-related colitis through microecological interventions are expected to revolutionize tumor immunotherapy.

## Gut microbiota modulation in the prevention and treatment of CRC

**Figure 2.**
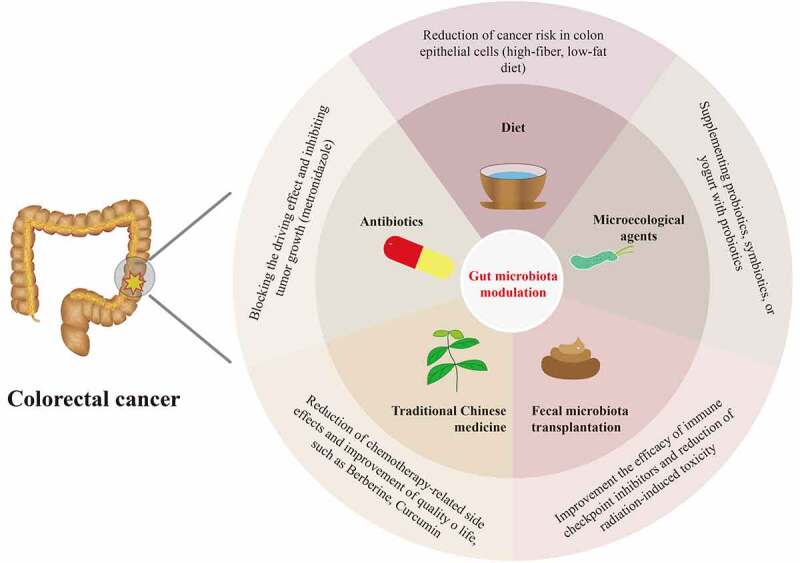
Overview of candidates for gut microbiota modulation for the prevention and treatment of colorectal cancer, including diet modulations, microecological agents, fecal microbiota transplantation, antibiotics, and traditional Chinese medicine.

Studies have shown that CRC could be prevented by the intestinal microbiota modulation such as diet interventions, probiotics supplement and antibiotics administration. Moreover, there is mounting evidence that several microecological agents, FMT, and TCM could influence the therapeutic effects and clinical outcomes of CRC by modulating the gut microbiota. Therefore, we discuss the promising gut microbiota modulators that are expected to be applied in the prevention and treatment of CRC (Figure 2).

### Diet modulations

Chinese medicine theory holds that “medical and edible food and medicine are from the same root,” and an old Chinese proverb says, “Diseases enter by the mouth.” Diet plays a central role in maintaining intestinal microecological homeostasis, and dietary factors influence CRC. Excessive intake of red meat and processed meat products can increase the risk of CRC, while a high-fiber diet helps prevent CRC.^50^ A study showed that the abundance of carbohydrate fermentation and butyrate-producing microorganisms in a native African population was higher; in contrast, more microorganisms related to BAs metabolism were found in African Americans.^123^ These differences are related to different dietary structures. The intestinal flora structure of African Americans changed significantly after taking the high-fiber, low-fat diet. CRC-related molecular markers were reduced in tissues, suggesting that the high-fiber, low-fat diet inhibits harmful intestinal bacteria, improves the abundance of beneficial bacteria, increases butyrate production and glycolysis capacity, reduces the synthesis of secondary BAs, and reduces the risk of colon epithelial cell carcinogenesis.^59^ Dietary factors affect CRC occurrence and progression by affecting intestinal microbiota and metabolites. Dietary interventions such as functional food supplements containing probiotics or other ingredients maintain the stability of the intestinal mucus layer and the homeostasis of gut microbiota-host interactions.^124^

### Microecological agents

The role of microecological agents such as probiotics, prebiotics, synbiotics, and even postbiotics in CRC is gradually being recognized. Probiotic products are subject to significant differences in strains, dosage forms, study populations, evaluation standards, and other factors at home and abroad, and the clinical application of probiotic preparations in China has its unique characteristics. The probiotics approved by the National Health Commission of China for use in humans mainly include *Lactobacillus*, *Bifidobacterium*, *Enterococcus*, *Streptococcus*, *Bacillus*, *Clostridium*, and *Yeast* at the genus levels.^125^

Microbiota modulators present a new vision for the prevention and the anti-tumor treatment of CRC. Evidence has suggested that high consumption of yogurt with probiotics moderately reduces the risk of CRA and CRC.^126,127^ Notably, both *L. casei* Zhang and *L. rhamnosus* Probio-M9 probiotic strains from China reduced the development of colon tumors in mice by improving intestinal microbial dysbiosis.^128,129^ Probiotics inhibit the development of CRC by increasing the abundance of probiotics, inhibiting various pathogenic bacteria, producing SCFAs, down-regulating the expression of pro-inflammatory factors, modulating the immune system, and promoting apoptosis.^130^ Supplementing probiotics, symbiotics, or yogurt with probiotics for those CRC patients receiving chemoradiotherapy or surgery could lower the complications, such as chemotherapy-related diarrhea/colitis, radiation enteritis, and postoperative adverse events.^131,132^ Preclinical studies showed that *Enterobacteriaceae*, *Bifidobacterium*, and *Akkermansia muciniphila* could improve the efficacy of CRC immunotherapy.^133^ Besides, *L. rhamnosus* Probio-M9 enhances the efficacy and responsiveness of anti-PD-1 immunotherapy, which may be a potential candidate for microecological agents.^134^ In the future, developing novel microecological agents in adjuvant anti-CRC therapy deserves further anticipation.

### FMT

The oldest description of FMT is from Chinese medicine. “Prescription Collection of Fifty-Two Diseases” recorded the use of “golden juice” to treat infectious diseases as early as the Western Zhou Dynasty of China (about the tenth century BC). Dr. Ge Hong, in the Eastern Jin Dynasty of China (about 1700 years ago), edited the “Prescription Collection of Emergency” and called the FMT preparation “yellow soup,” which was used to treat food poisoning and severe diarrhea.^135^ FMT is a critical way of modulating the imbalance of gut microbiota by transplanting functional fecal microflora of healthy individuals into the gastrointestinal tract of patients. FMT is primarily used for treating refractory or recurrent *C. difficile* infections, with a more than 90% cure rate. In addition, there are prospects for therapeutic application in inflammatory bowel disease, functional intestinal disorders, metabolic diseases, and rheumatoid immune diseases.^135–137^

FMT has become a research hotspot associated with cancer therapy, especially for enhancing the efficacy of ICIs, improving ICIs efficacy, drug resistance, and intestinal toxicity.^138,139^ Data regarding FMT in CRC are somewhat limited; however, a preclinical study showed that a mixture of 11 commensal bacteria enhanced the activity of ICIs and inhibited tumor growth in a mouse model of CRC.^122^ A study showed that FMT improved the drug resistance of melanoma patients to ICIs and restored the immunotherapeutic response, possibly related to the activation of T cell activity in tumors.^122,138^ Currently, many clinical studies are being performed to evaluate FMT for improving the efficacy and treatment sensitivity of ICIs. In addition, FMT modulates the intestinal toxicity of chemoradiotherapy and immunotherapy, including severe chemotherapy-related diarrhea or colitis, radiation enteritis, and ICI-related colitis.^112,140^ FMT has been used to treat refractory ICIs-associated colitis, and complete remission has been reported after single or multiple FMT.^141^ Preclinical studies showed that FMT alleviates severe diarrhea and intestinal mucositis associated with 5-FU and platinum in CRC mice.^140^ FMT improved diarrhea, abdominal pain, hematochezia, and other symptoms associated with radiation enteritis,^142^ suggesting the value of FMT in reducing radiation-induced toxicity and improving radiotherapy outcomes.^112^ Nevertheless, in-depth studies are needed before applying FMT to cancer therapy; definitions are needed for indications, donor screening, sample quality control, transplantation timing, and safety of FMT for CRC prevention and treatment.

### Antibiotics

Antibiotics have direct bactericidal or bacteriostatic effects on intestinal microflora; however, the relationship between antibacterial drugs and CRC is controversial. A large case-control study showed an association between antimicrobial agents and CRC.^143^ Theoretically, antibiotics might block its drive effect and inhibit tumor growth for *F. nucleatum*, *E. coli* sp., and other CRC-related driver bacteria. Studies found that *F. nucleatum* in primary lesions and liver metastases of CRC; the number of *F. nucleatum* in tumors decreased, and tumor growth slowed after treatment with metronidazole, suggesting that antimicrobials may have potential therapeutic value for *F. nucleatum* infection-related CRC.^101^ The influence of antibiotics on the gut microbiota and its role in CRC needs to be further studied.

### TCM

TCM has a long history of application in preventing and treating CRC as a predominant source of natural medicines and herbal products. In China, TCM has excellent potential to combat colorectal carcinogenesis while reducing the side effects associated with chemotherapy and improving the quality of life of CRC patients. TCM could inhibit CRC development by regulating CRC-related signaling pathways, such as PI3K/Akt, NF-κB, MAPK, and Wnt/β-catenin.^144^

Accumulating evidence suggests the fundamental role of the gut microbiota in the prevention and treatment of CRC through TCM, with the most frequently studied TCM ingredients being Berberine and Curcumin. A randomized double-blind controlled trial has demonstrated that Berberine effectively reduces the risk of CRA recurrence, suggesting that it is a promising agent for preventing and treating CRC.^145^ The specific role of gut microbiota in Berberine’s inhibition of CRC tumorigenesis has been elucidated in a set of studies.^146,147^ A recent investigation of the fecal microbiota of the CRC mouse model using 16S rRNA profiling identified a significant decrease in microbial richness without loss of diversity and an increase in the relative abundance of beneficial bacteria after Berberine treatment, accompanied by inhibition of several inflammatory and oncogenic pathways, such as NF-κB pathway.^147^ Preclinical studies have shown that Berberine inhibits CRC initiation and progression by regulating the intestinal microbiota and modulating the tumor microenvironment.^146^ Besides, Curcumin is identified as a well-tolerated chemotherapeutic adjuvant to oxaliplatin chemotherapy in patients with colorectal liver metastases. It mediates cell cycle arrest and ameliorates Treg-associated inflammation by regulating gut microbiota to exert anti-tumor effects.^148^ However, there is a lack of research data on the interactions between TCM and intestinal microbiota in CRC. Future research on the role of TCM in CRC prevention and treatment from the perspective of gut microbiota deserves in-depth study.

## Conclusion

In the past decades, the morbidity and mortality of CRC in China have increased, transitioning to developed countries such as Europe and the United States. A large population, unique genetics of multiple ethnic groups, variable environments, lifestyles, and dietary and gut microbiota structures are characteristics of China. Gut microbiota dysbiosis is associated with CRC progression. Some specific microbes contribute to CRC carcinogenesis, including *F. nucleatum*, pks^+^ *E. coli*, ETBF, and *P. micra*. However, the role of fungi and viruses in CRC needs further investigation. The microbial pathogenesis of CRC involves gut microbiota dysbiosis, invasion and colonization of pathogens, microbial-related metabolites, and virulence, which induce pro-inflammatory signaling pathways and exacerbate intestinal epithelial DNA damage, ultimately initiating CRC progression. However, high-quality in vitro and in vivo validation and in-depth evaluation are needed to clarify the gut microbiota-related mechanisms in the carcinogenesis of CRC. Early diagnosis and prediction by microbial biomarkers are promising research areas with significant clinical translational value. Integrating and optimizing intestinal microbiota-related biomarker panels with traditional clinical methods to improve CRC screening and diagnosis levels is promising and worth digging into. Gut microbiota interventions, including microecological agents and FMT, hold potential clinical applications to enhance drug efficacy, improve drug resistance, and reduce the severe intestinal toxicity associated with anti-tumor therapy of CRC. Moreover, TCM plays anti-tumor roles in CRC as an adjuvant treatment scheme in China. However, in-depth studies combined with the intestinal microbiota are still needed. Today, international cooperation is essential for searching for gut microbiota-related carcinogenic pathways, biomarkers, and interventional approaches to CRC.
